# Rising congenital syphilis amid declining perinatal HIV: a 7-year study from a tertiary medical center in Oregon

**DOI:** 10.1128/spectrum.02593-25

**Published:** 2026-01-06

**Authors:** Louise Vaz, Judith A. Guzman-Cottrill, Siouxzanna Downs, Rabeka Ali, Johanna B. Warren, Xuan Qin

**Affiliations:** 1 Department of Pediatrics, Doernbecher Children’s Hospital, Oregon Health & Science University 6684, Portland, Oregon, USA; 2 Department of Pathology and Laboratory Medicine, Oregon Health & Science University 6684, Portland, Oregon, USA; 3 Department of Family Medicine, Oregon Health & Science University 6684, Portland, Oregon, USA; University of Minnesota Twin Cities, Minneapolis, Minnesota, USA

**Keywords:** syphilis, HIV, congenital infections, prenatal, transmission, prevalence

## Abstract

We analyzed laboratory testing and positivity trends for syphilis and human immunodeficiency virus (HIV) to evaluate the effectiveness of public health policies and clinical practices. Laboratory data from unique patients were reviewed for syphilis (2018–2024) and HIV (2021–2024), stratified by age, gender, prenatal or newborn status, and county of residence in Oregon. Syphilis testing volumes reflected shifts in healthcare utilization before, during, and after the COVID-19 pandemic. HIV testing increased steadily from 6,627 patients in 2021 to more than 18,600 in 2024. Despite an average of ~10,000 syphilis tests annually, positivity rose from 1.8% in 2018 to 4.3% in 2024, peaking at 5.3% in 2021. In contrast, HIV positivity declined from 1.0% to 0.5% during the same period. Pediatric syphilis also increased—from 2 cases in 2018 to 16 in 2024—all attributed to maternal transmission. No new pediatric HIV cases were identified. Marked gender disparities were evident. Although females were tested at more than twice the rate of males, males were two to five times more likely to test positive for syphilis and two to seven times more likely for HIV. Geographically, cases clustered in densely populated counties with limited healthcare access, reflecting persistent inequities. Syphilis is highly treatable, and congenital cases are largely preventable with timely prenatal screening and treatment. Rising congenital incidence and increasing adult positivity highlight ongoing gaps in screening and prevention. These findings support urgent national action to require universal prenatal syphilis screening and expand risk-based testing for men and underserved populations.

**IMPORTANCE:**

This retrospective study evaluates laboratory-based syphilis and human immunodeficiency virus (HIV) testing outcomes at Oregon Health & Science University from 2018 to 2024, including more than 100,000 patient records. We report a troubling rise in congenital syphilis cases and late-trimester maternal diagnoses, despite existing prenatal screening protocols. In contrast, pediatric HIV was nearly absent, highlighting the impact of universal maternal screening and treatment. Our findings also emphasize persistent disparities by sex, age, and geography and call for a more robust, tiered prenatal syphilis screening policy, along with targeted partner testing. This work is highly relevant to clinicians, public health leaders, and policymakers focused on infectious disease prevention, maternal–child health, and health equity.

## INTRODUCTION

The United States has experienced a dramatic resurgence of syphilis cases in recent years, with a particularly alarming increase in congenital syphilis (1). According to the Centers for Disease Control and Prevention (CDC), syphilis rates have surged across all demographics (2). The rise in congenital syphilis cases underscores a critical gap in prenatal screening and treatment, highlighting the need for more stringent prenatal screening policies. When compared to human immunodeficiency virus (HIV), which has seen a substantial decline in perinatal transmission due to widespread prenatal testing and treatment programs, the syphilis epidemic presents an urgent public health challenge that requires immediate intervention (3, 4).

Syphilis, caused by *Treponema pallidum*, is a sexually transmitted infection that can lead to severe complications if left untreated. The disease has four stages—primary, secondary, latent, and tertiary—each presenting with varying clinical manifestations. When acquired during pregnancy, syphilis can be transmitted to the fetus, resulting in congenital syphilis, which can cause stillbirth, neonatal death, or lifelong health complications such as neurological impairment, bone deformities, and organ damage (5).

Despite the availability of effective screening and treatment options, congenital syphilis cases continue to rise. The CDC reported that in 2022, congenital syphilis cases had increased by over 30% compared to previous years, with hundreds of preventable neonatal deaths and severe health complications (3). The failure to identify and treat syphilis during pregnancy is often due to inadequate prenatal care, missed screening opportunities, and barriers to healthcare access (2).

Congenital infection poses additional challenges. Infants born to seropositive birthing parents require non-treponemal testing. A detailed algorithm incorporating both maternal and infant paired studies, as well as physical exam and maternal history, allows for a rubric of four pathways to ensure those potentially infected are treated: proven/highly probable syphilis, possible syphilis, syphilis less likely, and syphilis unlikely. Infants who fall in the first two categories are preferentially treated with parenteral aqueous or procaine penicillin G for 10 days. A one-time dose of intramuscular benzathine penicillin G injection is given when syphilis is less likely. No treatment is required for syphilis unlikely (6, 7).

This study summarizes 7 years of syphilis and HIV testing data from a major medical center in Portland, OR, to highlight disparities in screening, identify missed prevention opportunities, and inform recommendations for universal prenatal and targeted newborn syphilis screening.

## MATERIALS AND METHODS

### Population and data inclusion criteria

Laboratory test results from clinical diagnostic encounters were included for this analysis. All testing was conducted as part of routine patient care at Oregon Health & Science University and not for surveillance or research purposes. Data were stratified by age, sex, and county of residence in Oregon. A 7-year data set (2018–2024) for syphilis diagnostics and a 4-year data set (2021–2024) for HIV testing were analyzed.

Only fully confirmed clinical diagnostic results were included in the analysis. For each unique patient, the first recorded positive test (confirmed by reflexive multi-tier testing) was used. For patients who tested negative throughout the study period, the first negative result was used. This methodology ensured a standardized data set reflecting individual-level diagnostic encounters without duplicating results from follow-up testing. For the analysis conducted across sexes, analysis was limited to males and females. Reported sexes that were “unknown” or “unspecified” were removed from the analysis due to low volumes (<5 total tests over the reporting period).

Age groups are developed based on appropriate test algorithms, clinical services, and epidemiology conventions. Syphilis diagnostic testing and findings were analyzed in the following age groups: newborns (≤28 days), infants (>28 days to ≤18 months), and children (>18 months to ≤13 years), adolescents and adults (>21 to ≤65 years), and seniors (≥65 years). Pregnant patients were identified by the total number of births that occurred in the hospital system where there were also records of prenatal care. Patients without records of active prenatal care were excluded from the analysis due to the inability to accurately determine if the patient would have received syphilis screening while receiving care at another service.

### Laboratory tests and diagnostic algorithms

Prior to March 2024, all syphilis diagnostic testing followed the traditional algorithm, beginning with a rapid plasma reagin (RPR, BD Macro-Vue RPR Card Tests) screening, followed by RPR titer and *Treponema pallidum* particle agglutination (TP-PA) testing. Starting in March 2024, following the CDC guidelines (8), the traditional algorithm remained in use for patients ≤18 months of age and for individuals of any age with a known history of syphilis. For all other patients over 18 months, a reverse algorithm was implemented, beginning with the Alinity i Syphilis TP Assay (Abbott), followed by RPR**,** RPR titer, and TP-PA confirmation (8). The Alinity i Syphilis TP Assay is a chemiluminescent microparticle immunoassay designed for the qualitative detection of IgG and IgM antibodies against *Treponema pallidum* in human serum or plasma using the Alinity i Analyzer (Abbott).

In parallel, all HIV diagnostic testing followed the CDC/APHL-recommended laboratory testing algorithm (9). Due to incomplete digital test record integration prior to 2021 as a result of the HIV three-tier tests (i.e., HIV antigen/antibody screen, confirmatory antibody differential test, and HIV RNA quantitative PCR) not being performed at the on-site laboratory, HIV test data before 2021 were excluded from this analysis.

### Data analysis

Data visualization and analysis were performed in Tableau Desktop version 2023.3. Analysis included an initial query of EPIC electronic medical records via Structured Query Language. Data validation and chart review of positive cases, especially of pediatric patients, was conducted to ensure accuracy before data were loaded into Tableau for further analysis.

### Statistical analysis

Analysis was conducted in Microsoft Excel and validated using R version 4.40. Three tests were conducted using a chi-square test of independence, one across sexes, one across five age categories, and one for the maternal testing based on which trimester care was established for the pregnancy episode. Tests across sexes and ages were conducted for both HIV and syphilis, and the tests for maternal positivity were for syphilis only.

## RESULTS

### Trends in testing volume and positivity rates

Laboratory testing data from 2018 to 2024 revealed important trends to inform clinical practice and public health strategy. A 7-year data set for syphilis testing and a 4-year data set for HIV testing were analyzed.

**Fig 1:**
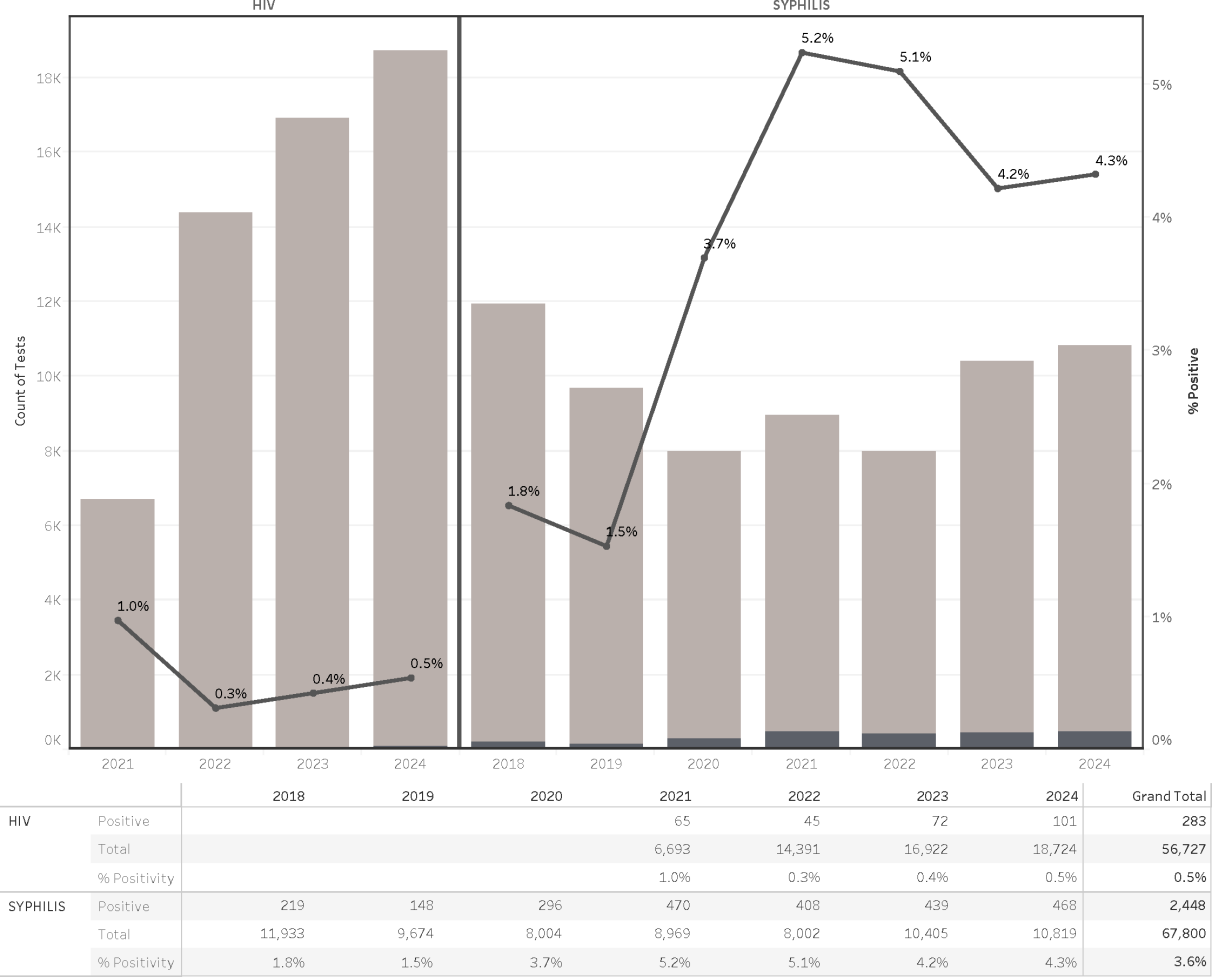
Trends of syphilis and HIV diagnostic tests at a major medical center in Oregon.

Syphilis testing volumes remained relatively stable over the 7-year period, ranging annually from approximately 8,000 to nearly 12,000 patients. In contrast, HIV testing volume exhibited a marked and consistent increase over time, more than doubling from 6,693 tests in 2021 to 18,724 in 2024 (Fig. 1).

Trends in positivity rates diverged between the two infections. Syphilis positivity increased over time, rising from 1.8% in 2018 to 4.3% in 2024, with a peak of 5.2% in 2021. In contrast, HIV positivity showed a gradual decline, decreasing from 1.0% in 2021 to 0.5% in 2024, despite the growing testing volume. These patterns suggest distinct epidemiologic dynamics for each infection, with syphilis demonstrating a growing burden and HIV potentially reflecting improved screening outreach or earlier diagnosis.

**Fig 2:**
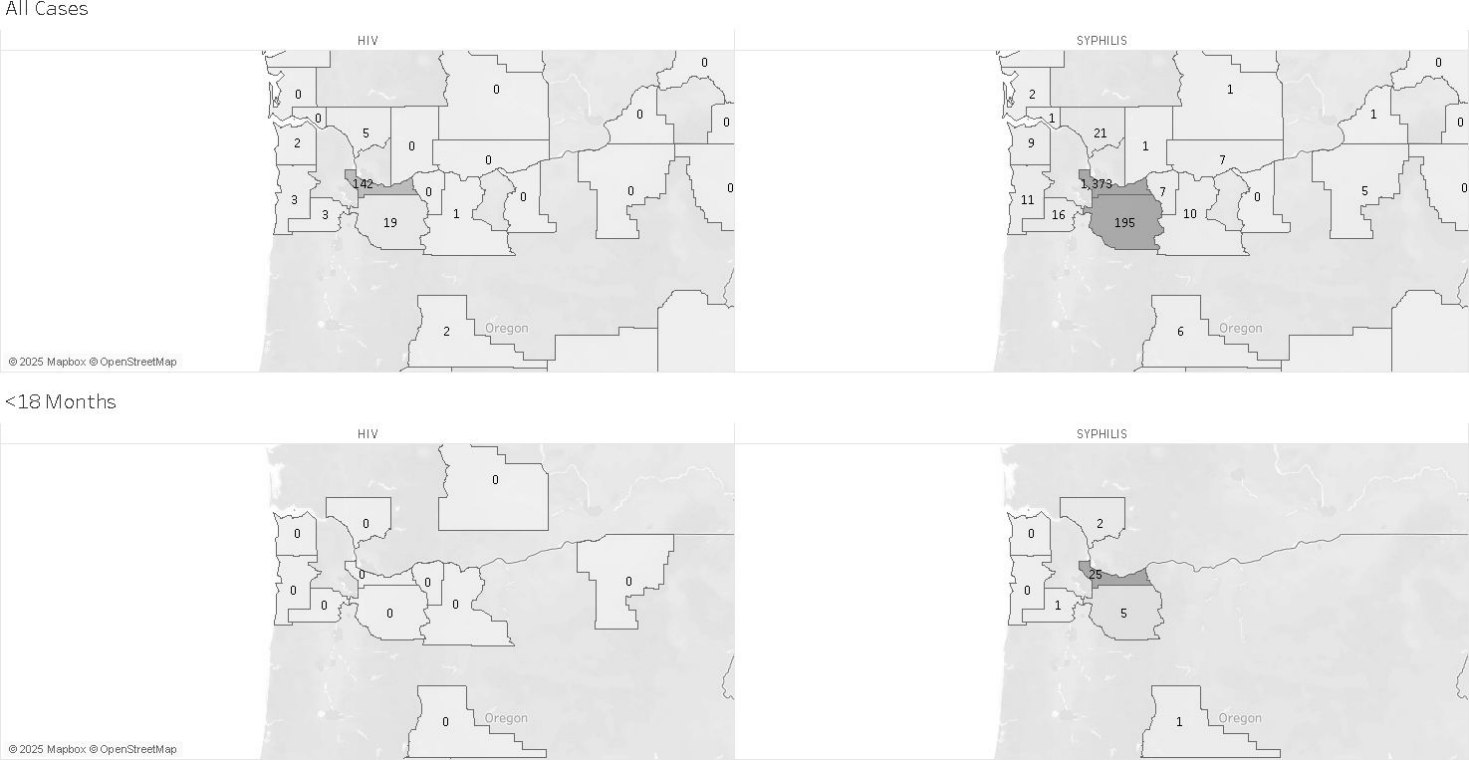
Geographic distribution of new HIV (2021–2024) and syphilis cases (2018–2024) in Oregon and neighboring Washington State by county.

Our geographic analysis revealed a pronounced concentration of both HIV and syphilis cases in Oregon’s most populous urban county. The top panels in Fig. 2 show that the majority of total reported cases across all age and sex groups occurred in this single county, Multnomah County. Notably, the bottom panels demonstrate that newborn and infant HIV and syphilis cases were similarly clustered, with the Portland metropolitan area representing the epicenter of perinatal disease burden. These spatial patterns suggest strong geographic correlations between community-level risk and pediatric case detection.

### Gender disparities in testing volume and positivity rates

**Fig 3:**
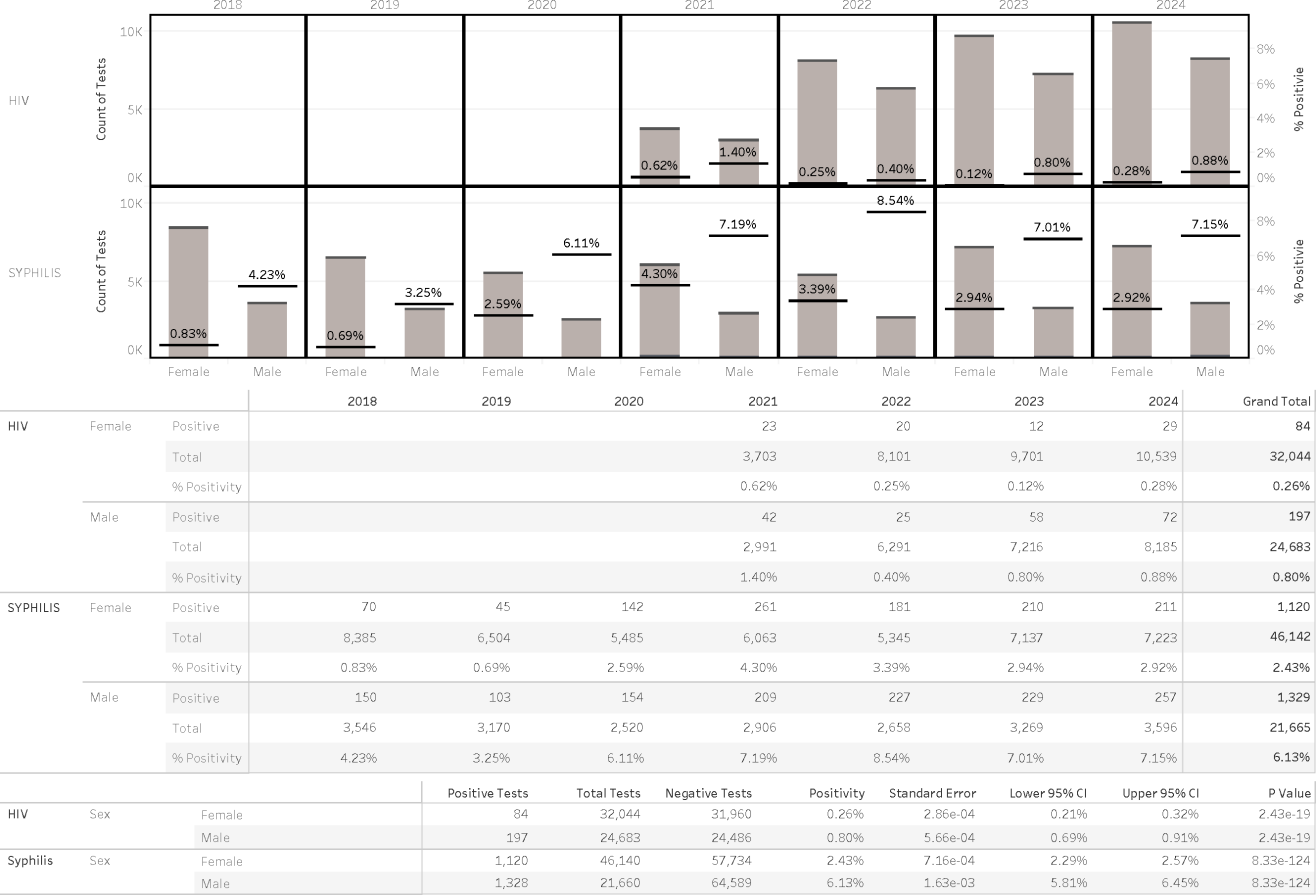
Trends of syphilis and HIV diagnostic tests between males and females.

For both HIV and syphilis testing, a clear gender disparity was observed: males were tested far less frequently than females yet were significantly more likely to test positive (Fig. 3).

The multi-year data reveal consistent and statistically significant sex-based differences in test positivity for both HIV and syphilis, though the time frames of available data differ. HIV data (2021–2024) show that males had consistently higher positivity rates than females each year, with male rates ranging from 0.40% to 1.40% and female rates ranging from 0.12% to 0.62%. In aggregate, HIV positivity was 0.80% in males vs 0.26% in females (*P* = 2.12 × 10⁻¹⁹).

Syphilis data (2018–2024) similarly show a sustained and marked disparity. Male positivity rates rose from 4.23% in 2018 to 7.15% in 2025, while female rates remained relatively stable and substantially lower, ranging from 0.83 in 2018 to a peak of 4.3% in 2021 and to 2.92% in 2024. Overall, syphilis positivity was 6.13% in males vs 2.43% in females (*P* = 8.33 × 10⁻¹²⁴).

Female testing volumes exceeded those of males, 1.3-fold for HIV (31,957 vs 24,575 tests) and 2.1-fold for syphilis (46,140 vs 21,660 tests), yet males contributed disproportionately to the total number of positive results (Fig. 3).

### Disease incidence by age distribution

#### HIV positivity by age group

**Fig 4:**
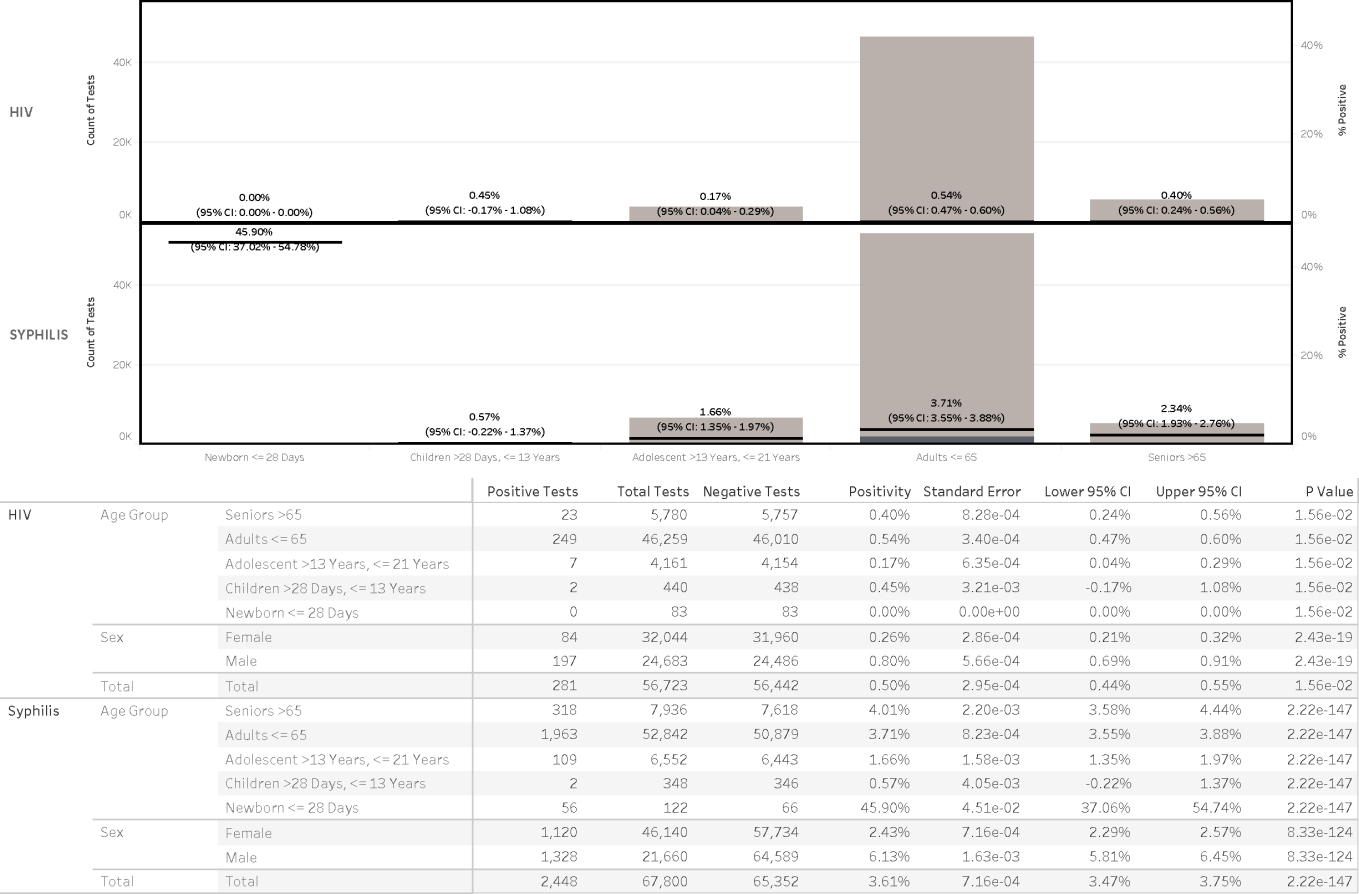
Age distribution of patients undergoing syphilis and HIV testing.

Across all age groups, 56,727 HIV tests were performed with an overall positivity rate of 0.50% in the past 5 years (Fig. 4). Adults aged ≤65 years accounted for the majority of HIV-positive cases (*n* = 249) with a positivity rate of 0.54% (95% CI, 0.47%–0.60%) (Fig. 4). Seniors aged >65 years were tested far less frequently (*n* = 5,780) vs adults aged ≤65 (*n* = 46,259); however, seniors aged >65 years of age had a positivity rate of 0.40% (95% CI, 0.24%–0.56%), which is not negligible and warrants inclusion in clinical screening strategies. Adolescents aged 13–21 years showed lower positivity (0.17%), whereas children aged >28 days to 13 years showed a higher rate of 0.45%, which was influenced by two children through international adoption. No positive HIV tests were detected among newborns aged ≤28 days (0 of 83). Although testing was concentrated in younger adults, statistically significant differences in positivity rates were observed across age groups (*P* = 1.80 × 10⁻²), underscoring meaningful positivity even among less frequently tested children and seniors.

**Fig 5:**
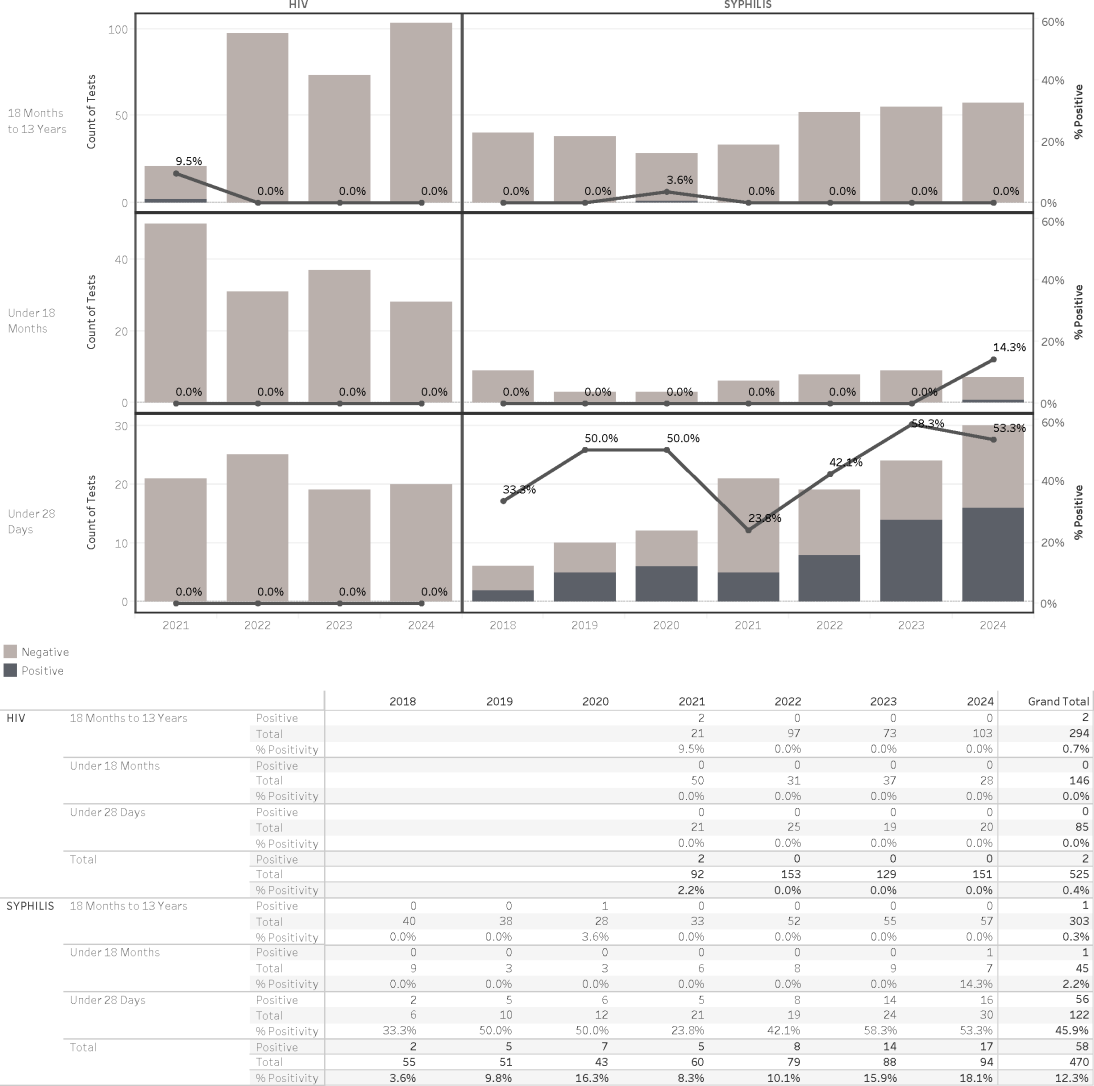
Syphilis diagnostic testing and findings in newborns, infants, and children—trends and positive rates.

#### Syphilis positivity by age group

Among 67,800 syphilis tests, the overall positivity rate was 3.61% over the past 7 years. Adults aged ≤65 years again comprised the majority of the tests (*n* = 52,842 or 78%) with a positivity rate of 3.71% (95% CI, 3.55%–3.88%) (Fig. 4). Seniors aged >65 years, despite a relatively low testing volume (*n* = 7,936 or 12%), had a slightly higher positivity rate of 4.01% (95% CI, 3.58%–4.44%), highlighting a potentially under-recognized burden in this age group. Adolescents aged 13–21 years and children aged >28 days to 13 years showed lower positivity rates of 1.66% and 0.57%, respectively. Notably, newborns aged ≤28 days demonstrated an extraordinarily high positivity rate of 45.90% (56 of 122 tested; 95% CI, 37.06%–54.74%) that is consistent with its targeted testing policies and practice, but the 7-year upward case trend is strongly suggestive of congenital syphilis and signaling gaps in maternal screening or treatment.

### Comparison of syphilis and HIV findings in newborns, infants, and children: implications for prenatal screening

Syphilis and HIV testing among newborns and children remained low throughout the study period, consistent with targeted, risk-based diagnostic practices. Syphilis testing in newborns (≤28 days old) increased modestly over the 7-year period, from 6 tests in 2018 to 30 in 2024 (Fig. 5). The median age at syphilis diagnosis was 3 days (range: days 1–14, data not shown). One notable case in 2024 involved a 4-month-old adopted infant who had tested positive and received treatment at birth elsewhere and was later reevaluated after adoption under our pediatric care. The biological mother of the infant had an incomplete record of prenatal care. In addition, a 13-year-old female was diagnosed with syphilis in 2020; after a comprehensive clinical and social work review, child sexual abuse was formally ruled out (Fig. 5).

In contrast, HIV testing among newborns remained infrequent, reflecting the effectiveness of routine prenatal screening and maternal prophylaxis. A total of 85 newborns were tested for HIV across the study period, with no positive results identified between 2021 and 2024. Similarly, among infants and young children tested based on risk or clinical indication, HIV positivity remained rare. Two cases of pediatric HIV were detected in 2021 among 7- and 10-year-old internationally adopted children. Both had known congenital HIV diagnoses and were identified upon reestablishment of care after immigration (Fig. 5).

**Fig 6:**
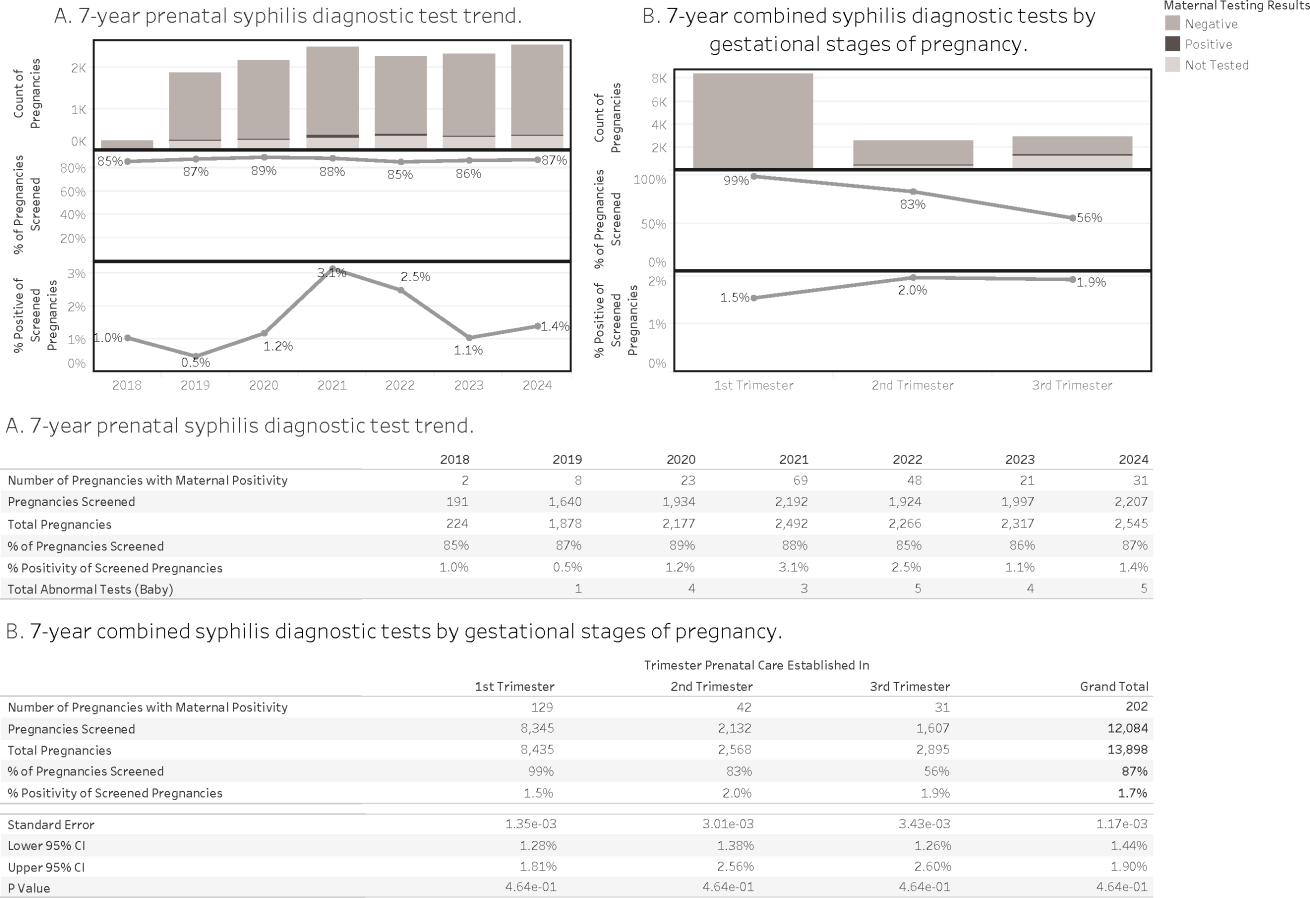
Syphilis diagnostic testing and findings in prenatal care (A and B).

Our analysis of prenatal syphilis screening across 13,889 pregnancies over a 7-year period revealed key trends relevant to congenital case prevention (Fig. 6). Overall, 87% of pregnancies were screened for syphilis, with annual screening rates ranging from 85% to 89%. However, among pregnancies with confirmed maternal positivity (*n* = 202), screening frequently occurred late in gestation: only 64% of these positive cases were identified in the first trimester, while 21% were first screened in the second trimester and 15% not until the third. Positivity rates among screened pregnancies increased progressively by trimester, from 1.5% in the first trimester to 2.0% and 1.9% in the second and third trimesters, respectively.

## DISCUSSION

This study provides a comprehensive assessment of syphilis and HIV testing trends and disparities across pediatric, perinatal, and adult populations over a 7-year period. HIV incidence remained consistently low, with no pediatric HIV cases identified in the past 4 years, reflecting the success of universal prenatal screening and standardized antiretroviral protocols. In contrast, syphilis incidence rose steadily from 1.8% in 2018 to 4.3% in 2024, accompanied by an alarming increase in congenital syphilis—from 2 cases in 2018 to 16 in 2024. Nearly half of tested newborns were syphilis positive, often diagnosed within the first 2 weeks of life.

Geospatial analysis revealed a pronounced concentration of both HIV and syphilis cases in Oregon’s urban core, particularly in the Portland metropolitan area. This clustering aligns with known structural inequities, including housing instability, limited access to prenatal care, and under-resourced healthcare infrastructure, which collectively contribute to elevated disease burden. These urban hotspots represent a syndemic landscape where social determinants of health intersect with infectious disease transmission, disproportionately affecting underserved communities.

Further disparities were evident across age and sex groups. Males, despite being tested far less frequently than females, exhibited significantly higher positivity rates, approximately two to five times higher for syphilis and two to seven times higher for HIV. This disparity underscores missed opportunities for targeted screening and intervention. Additionally, older adults (>65 years), often excluded from routine STI screening, demonstrated non-negligible positivity rates, challenging prevailing assumptions about risk distribution and highlighting the need for more inclusive testing strategies across the lifespan.

Our prenatal data showed that although on average 87% of pregnancies underwent syphilis screening, nearly one-third of maternal syphilis cases were not identified until the second or third trimester. These delays undermine prevention and allow vertical transmission to occur. Incomplete documentation and fragmented care further limited follow-up and treatment. Importantly, reinfection cannot be ruled out and likely contributes to congenital cases. Reinfection may occur not only during pregnancy but across all age and gender groups, driven in part by untreated or undertested sexual partners.

Notably, undertested male partners likely play a key role in sustaining the cyclical nature of syphilis transmission. The persistently low testing volume among men, despite higher positivity rates, suggests significant missed opportunities for intervention. Routine screening of male partners of all ages, particularly those of pregnant individuals, must be prioritized. Without identifying and treating partners, maternal reinfection and congenital transmission will remain difficult to prevent, regardless of how timely or thorough the initial prenatal screening.

These findings support the urgent need for a universal, tiered prenatal syphilis screening policy: testing at the first prenatal visit, repeat screening in the second and the third trimester, and at the time of delivery in cases of ongoing risk or incomplete history. Newborns with unknown or inadequately screened maternal status should undergo point-of-care RPR testing. Early diagnosis and timely treatment remain highly effective (10), but their impact depends on access, continuity of care, and partner management.

The success of perinatal HIV prevention offers a clear roadmap. Routine maternal HIV testing and universal access to antiretroviral therapy have driven vertical transmission rates in the United States to below 1% (Panel on Treatment of HIV in Pregnancy, updated 12 June 2024). As shown in prior studies (3, 4), systematic, population-wide screening and follow-up, rather than risk-based strategies, are essential for equitable prevention.

Unlike HIV, which requires lifelong antiretroviral management (11), an episode of syphilis is generally curable with penicillin treatment. However, the absence of structured monitoring after treatment, especially in high-risk populations, makes reinfection more likely. Variable practices in follow-up serologic testing, incomplete partner treatment, and lack of coordinated prevention strategies all contribute to this cycle. The relative simplicity of syphilis treatment should not overshadow the complex public health systems needed to sustain prevention.

This study has several limitations. First, only the first confirmed diagnostic result per patient was included, either the first positive or, if consistently negative, the first negative, over 7 years for syphilis and 4 years for HIV. This approach excluded follow-up testing and may underestimate reinfection or delayed diagnoses. Second, maternal records for syphilis-positive newborns were not reviewed for disease stage, treatment response, or timing of reinfection. Third, detailed information on prenatal care access, adherence, and care coordination between maternal and newborn management was not available, which limited assessment of system-level failures. Finally, the absence of standardized policies for partner identification, treatment, and monitoring during pregnancy constrained our ability to evaluate the impact of partner management on congenital syphilis prevention.

In conclusion, congenital syphilis remains a preventable yet escalating public health crisis and a stark indicator of systemic failures in maternal–child health. Reversing this trend requires universal, repeated, and inclusive screening strategies encompassing pregnant individuals, their partners, and at-risk populations of all ages and sexes, paired with robust partner management, vigilant longitudinal follow-up of known positives and high-risk individuals, and targeted urban interventions to break the cycle of reinfection. The recent recommendation (13 May 2025) from the U.S. Preventive Services Task Force reinforces the urgency of universal prenatal screening (12). Expanding partner screening, particularly for asymptomatic male partners, remains critical, yet this essential component of CDC guidance continues to be underutilized in most prenatal care settings.
